# 

**Published:** 1898-12

**Authors:** 


					DECEMBER, 1898.
THE
AMERICAN JOURNAL
?W' O F w?
DENTAL SCIENCE.
EDITED
B
F. J. S. GORGAS, M.iLp^ D- D- S.
RICHARD GRADY, M. D? D. D. S.
Vol. 33.
THIRD SERIES
No. 8.
BALTIMORE!
SNOWDEN & COWMAN MFG. C O , 9 W. Fayette St.
LONDON!
TRUBNER & CO., 60 Paternoster Row.
Entered at the Post Office at Baltimore, Md? as Second-class Matter.
IPKYKBLE IN RDVKNCE.
PUBLISHED MONTHLY
l$2.j50i?ER HNNUM.

				

